# Clomiphene Associated Inferior STEMI in a Young Female due to Right Coronary Artery Dissection

**DOI:** 10.1155/2017/4747831

**Published:** 2017-05-16

**Authors:** Feras Husain Abuzeyad, Eltigani Seedahmed Ibnaouf, Mudhaffar Al Farras

**Affiliations:** Emergency Department, King Hamad University Hospital, Busaiteen, Bahrain

## Abstract

Nonatherosclerotic spontaneous coronary artery dissection (NA-SCAD) is an uncommon cause of myocardial infarction. It most commonly affects females in the perimenopausal age. NA-SCAD has been associated with many predisposing factors including pregnancy and hormonal therapy for both contraception and ovulation induction. The presented case is a previously healthy 41-year-old woman diagnosed with inferior ST-elevation myocardial infarction due to right descending coronary artery dissection associated with recent use of clomiphene monotherapy for ovulation induction (a previously fertile woman), which was not previously reported.* Learning Objectives*. Emergency physicians (EPs) should be aware about NA-SCAD as a cause of acute coronary syndrome (ACS) especially in perimenopausal women even with no risk factors. NA-SCAD occurs more commonly in the postpartum period and in females following hormonal therapy. Here, clomiphene monotherapy was reported as a possible contributing factor to NA-SCAD. Guidelines for NA-SCAD management are not up to date, and EPs should avoid some interference before the final diagnosis of the cause of myocardial infarction.

## 1. Introduction

NA-SCAD is a poorly understood cause of acute myocardial infarction and sudden cardiac death. It is associated with nearly 40% of acute coronary syndromes in female patients under the age of 50 years, and 50% of NA-SCADs present as ST-segment-elevation myocardial infarction, and 25% of cases present with multiple vessels disease [1, 2]. More knowledge about NA-SCAD will help in the accurate diagnosis and proper treatment especially for EPs since proper and timely intervention can be lifesaving with fewer complications.

## 2. Case Report

**Figure 1:**
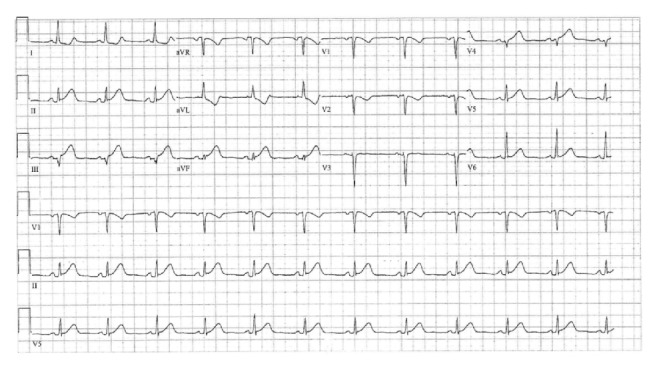
The initial ECG at presentation with active chest pain.

**Figure 2:**
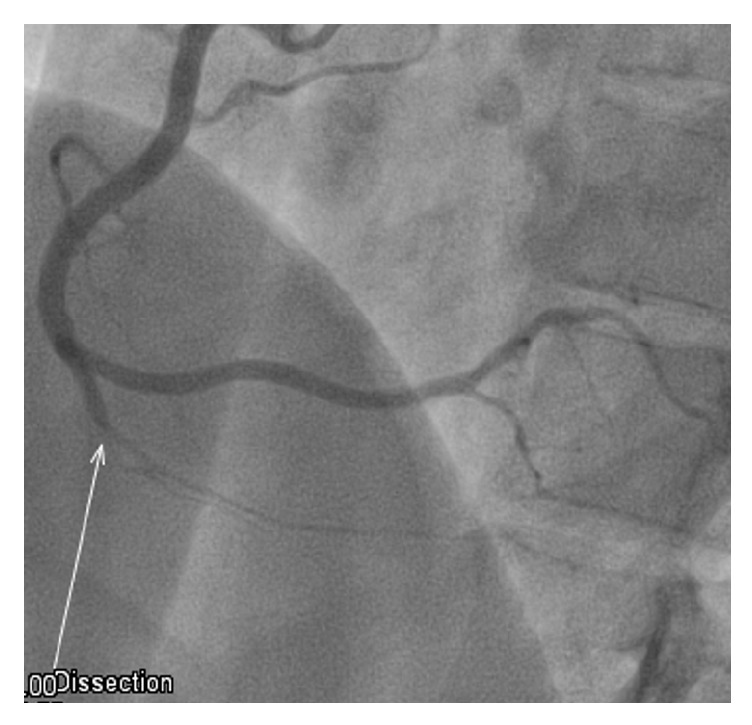
Coronary angiogram showing right descending coronary artery dissection.

A 41-year-old woman presented to the Emergency Medicine Department (EMD) with a sudden-onset left side chest pain of less than one hour in duration that started at rest. The pain was severe and radiated to the left shoulder. This was accompanied with symptoms of shortness of breath and sweating but no nausea, fever, cough, or similar episodes in the past. There was no significant history of coronary artery disease or any other illness in the family. She denied smoking tobacco, drinking alcohol, or illicit drug use. She gave a history of recent clomiphene citrate tablet use for ovulation induction which started 5 days earlier with no other hormonal therapy. In the emergency department, she was hemodynamically stable and her physical examination was within normal limits. Electrocardiogram (ECG) showed ST-segment elevation in the inferior leads (Figure 1). No prior ECG was available for comparison. Immediate treatment with morphine for pain control, aspirin 300 mg orally, and clopidogrel 600 mg loading oral dose was given along with nitrate sublingual tablet and then heparin loading with 5000 IUs was started. Direct cardiology consultation was done according to the King Hamad University Hospital EMD Chest Pain Policy. The cardiologist on call directly started her on thrombolytic therapy and then contacted the well established cardiac center in the Bahrain Defense Force-Medical Service Hospital (BDF-MSH) for emergency percutaneous coronary intervention (PCI). Her cardiac markers and all other initial basic laboratory investigations were negative and within normal ranges, respectively. She underwent PCI which showed right posterior descending artery dissection extending from the proximal to the distal segment with Thrombolysis in Myocardial Infarction (TIMI) angiographic flow grade III (Figure 2). All other coronaries were patent with TIMI III flow and no atherosclerotic changes. No stenting was done. Finally, the patient did well and was discharged from the cardiac center with conservative treatment including both aspirin and clopidogrel with follow-up appointment and no further assessment or investigations were done.

## 3. Discussion

NA-SCAD is linked to many precipitating factors that include fibromuscular dysplasia (FMD), pregnancy, systemic inflammatory diseases, hormonal therapy, multiparity, and cardiocirculatory stress, and about 20% of cases were idiopathic [1, 3]. In recent accounts of series, the true prevalence of NA-SCAD is about 1% to 4% of ACS overall, depending on the source [4]. NA-SCAD usually affects young female below 50 years of age without risk factors for atherosclerosis. The average age is 42 years and 80% of the patients affected are females. 20% to 25% of cases occur in the peripartum period [1]. There is a lack of certainty in regard to the causes, recurrence, and prognosis in addition to ideal management plans. However, there is a considerable increase in research recently due to social media and easy finding of affected patients [5].

Treatment with clomiphene in our patient of the study is assessed to be a likely causative factor to her dissection, an association which was reported once before in an infertile woman treated with gonadotropin injectable therapy along with clomiphene for multiple rounds before the development of NA-SCAD [6]. Clomiphene is a selective estrogen receptor modulator (SERM) that stops the feedback inhibition of the pituitary and hypothalamus, hence leading to increased production of estrogen by the ovaries. Clomiphene has unknown effects in the coronary arteries. Clomiphene citrate has been reported in association with different vascular conditions like myocardial infarction [7].

NA-SCAD is usually diagnosed by coronary angiography, which shows the false lumen and area of obstruction in the affected coronary vessel. CT angiography is recommended in inconclusive cases. Additional diagnostic tools include intravascular ultrasonography (IVUS), optical coherence tomography (OCT), and coronary computed tomography angiography (CCTA). Some studies found that OCT is more sensitive than IVUS as it illustrates more information about the dissection. Coronary angiography unfortunately has limitations for the diagnosis of NA-SCAD [8–10].

There are no clear guidelines for the acute management of NA-SCAD and early diagnosis is crucial for management, because the blind use of medications we use in acute coronary syndrome may lead to serious complications. For ACS, the initial emergency management is based on clinical symptoms, ECG findings, and laboratory results and should be initiated as soon as possible unless there is a contraindication.

Any early intervention or subsequent treatment depends on the TIMI findings and NA-SCAD extension and location. Aspirin is recommended for acute and long-term NA-SCAD treatment. Clopidogrel (or other P2Y12 antagonists) is of uncertain benefit in acute settings. Since intimal tear is prothrombotic, the use of dual antiplatelet therapy with aspirin in acute phase and clopidogrel for one year is recommended because it will reduce the narrowing of the lumen [10]. Other medications like GPIIb/IIIa inhibitors are not studied and are not routinely used because of bleeding risk and dissection extension possibility. Beta blockers on the other hand are recommended in acute settings as well as for long-term therapy. Nitroglycerin is useful in relieving ischemic symptoms from vasospasm during acute presentation [9].

The anticoagulation use for NA-SCAD is debated between the benefits of dissolving the overlying clot thrombus with improving lumen patency and the risk of dissection extension. However, there is consensus that heparin which is usually started for ACS presentations in the emergency department is discontinued once the diagnosis of NA-SCAD is established [10].

Regarding thrombolytic therapy, it is important to be avoided in NA-SCAD as there are reports of clinical deterioration caused by dissection extension. However, in areas where primary PCI is not available or the patient cannot be transferred to a cardiac catheter center, thrombolysis should not be avoided for ST-elevation MI patients as the overall incidence of thrombotic occlusion is more common than NA-SCAD [9, 10]. On the other hand, statins use for NA-SCAD has not been evaluated, and hence they can be used only for patients known to have dyslipidemia [9].

The revascularization interventions option depends on the patient clinical status and the coronary artery anatomy in question. Patients who show signs of clinical instability, ongoing symptoms, or ST elevation need to undergo PCI, especially when the culprit lesion of dissection is affecting the major coronary artery with considerable infarct size. When the dissected artery segment is distal or of small caliber or when the dissection is extensive, stenting may not be practical. Major intervention with coronary artery bypass graft is recommended if the left main coronary artery is involved, if multiple vessels are involved, or if the patient shows hemodynamic instability or is actively symptomatic [9, 11].

The overall mortality in hospital is not high; however, one of the problems of NA-SCAD is the recurrence of the dissection and further major cardiac events which highlight the need for close follow-up [1, 10]. In patients with NA-SCAD aggressive risk factor modification and medical therapy should be initiated. It is important to counsel female patients with NA-SCAD to use nonhormonal contraception and to avoid pregnancy as the risks of recurrence and complications of NA-SCAD are high [11]. In addition, screening for FMD especially in women is of paramount importance.

In conclusion, NA-SCAD is a rare disease of young adult women and presents acutely with sudden cardiac death, chest pain, ACS, and malignant ventricular arrhythmia. A number of different predisposing factors have been reported in the literature, including ovulation induction therapy with clomiphene, which we believe predisposed our patient to NA-SCAD. Once the patient survives the acute episode, the prognosis is fairly good. In general, treatment should be individualized, as there is no specific guideline on how to manage these patients. An aggressive strategy should be pursued if a large territory is involved or the patient is hemodynamically unstable or actively symptomatic. On long-term follow-up, these patients are at risk of recurrence, especially women. Therefore, it is important to counsel women against pregnancy, which is an important risk factor.
